# Asking Sensitive Questions Using the Randomized Response Approach in Public Health Research: An Empirical Study on the Factors of Illegal Waste Disposal

**DOI:** 10.3390/ijerph16060970

**Published:** 2019-03-18

**Authors:** Andy C. Y. Chong, Amanda M. Y. Chu, Mike K. P. So, Ray S. W. Chung

**Affiliations:** 1 School of Nursing and Health Studies, The Open University of Hong Kong, Ho Man Tin, Hong Kong; achong@ouhk.edu.hk; 2 Department of Social Sciences, The Education University of Hong Kong, Tai Po, Hong Kong; 3 Department of Information Systems, Business Statistics and Operations Management, The Hong Kong University of Science and Technology, Clear Water Bay, Hong Kong; immkpso@ust.hk; 4 Division of Environment and Sustainability, The Hong Kong University of Science and Technology, Clear Water Bay, Hong Kong; swchungaa@connect.ust.hk

**Keywords:** randomized response technique, sensitive question, unrelated question design, online survey, theory of planned behavior, general deterrence theory, solid waste charging scheme

## Abstract

A survey study is a research method commonly used to quantify population characteristics in biostatistics and public health research, two fields that often involve sensitive questions. However, if answering sensitive questions could cause social undesirability, respondents may not provide honest responses to questions that are asked directly. To mitigate the response distortion arising from dishonest answers to sensitive questions, the randomized response technique (RRT) is a useful and effective statistical method. However, research has seldom addressed how to apply the RRT in public health research using an online survey with multiple sensitive questions. Thus, we help fill this research gap by employing an innovative unrelated question design method. To illustrate how the RRT can be implemented in a multivariate analysis setting, we conducted a survey study to examine the factors affecting the intention of illegal waste disposal. This study demonstrates an application of the RRT to investigate the factors affecting people’s intention of illegal waste disposal. The potential factors of the intention were adopted from the theory of planned behavior and the general deterrence theory, and a self-administered online questionnaire was employed to collect data. Using the RRT, a covariance matrix was extracted for examining the hypothesized model via structural equation modeling. The survey results show that people’s attitude toward the behavior and their perceived behavioral control significantly positively affect their intention. This paper is useful for showing researchers and policymakers how to conduct surveys in environmental or public health related research that involves multiple sensitive questions.

## 1. Introduction 

A survey study is a conventional research method that is used to quantify population characteristics in biostatistics and public health research [1]. In health and environmental research, individuals’ behaviors and beliefs are usually assessed through a survey. Asking sensitive questions is sometimes inevitable in survey studies, but responses from interviewees may be distorted due to their desire to avoid social undesirability (embarrassment or the threat of disclosure). To mitigate the response distortion arising from evasive answers to sensitive questions, the randomized response technique (RRT) is a useful and effective statistical method for reducing such potential biases.

The RRT was first introduced by Warner [2] and subsequently was modified by Greenberg et al. [3] using an unrelated question design (UQD). To apply the RRT, respondents are assigned to answer either a sensitive question on the topic of interest, or an unrelated question, with the choice being determined by a privately generated outcome of randomization. The assignment results are not disclosed, and even the researchers do not know which question the respondent has actually answered. By undergoing this procedure, respondents are expected to be more confident in the anonymity of their responses and thus are expected to give trustworthy responses to sensitive questions.

Due to its feasibility in reducing the potential bias caused by sensitive questions, the RRT is applicable in studies on a wide range of sensitive topics in public health related research. For example, Kirtadze et al. [4] applied the RRT to estimate the prevalence of drug use, and Schröter et al. [5] used the RRT to assess the prevalence of doping in recreational triathletes. Deviating from the topic of substance use, Chen et al. [6] used the RRT to investigate commercial sexual behavior. In addition, Gingerich [7] employed the RRT to infer corruption phenomena. Researchers have tended to apply the RRT in a single dichotomous question in a survey. Moreover, as Blair et al. [8] (p.1304) addressed, the number of studies applying the RRT method is still “surprisingly few” in empirical research. Therefore, this paper aims to utilize the RRT in a multivariate analysis setting and to demonstrate its application in environmental research while riding on the popularity of the use of digital devices. The context of illegal waste disposal is used to illustrate the RRT procedure.

### 1.1. Illegal Waste Disposal

Illegal waste disposal is a global environmental problem. Its negative consequences are also a threat to the health of individuals and to public health in general [9]. Specifically, in Hong Kong, a massive amount of waste is generated daily. According to the Hong Kong Environmental Protection Department [10], there are 15,332 tons of solid waste disposed at landfills each day, while more than 60% is municipal solid waste. Additionally, Hong Kong citizens dispose of 1.41 kg of municipal solid waste per day, an amount much higher than in many other cities and countries, such as Taiwan (0.92 kg [11]), Seoul (0.95 kg [12]), and Japan (0.94 kg [13]). Therefore, the Hong Kong government proposes to institute a quantity-based charging scheme for municipal solid waste, which would be applied to most residential buildings. Such a scheme could be an effective method for waste reduction [14,15,16].

Nonetheless, based on the experiences from other countries [17,18], it is expected that the implementation of a waste charging scheme could increase the incidence of illegal waste disposal. In other words, households may discharge waste through unauthorized channels to evade the charge. Illegal waste disposal imposes huge damage to the environment and to society by degrading the environment, lowering the value of public lands and surrounding properties, impacting the visual amenities of lands, attracting further illegal waste disposal, and imposing extra clean-up costs on the government [19]. Besides causing environmental and economic crises, illegal waste disposal can also increase the incidence of cancer and the rate of childhood mortality and malformation, according to other relevant studies [9].

Before the government launches a waste charging scheme, however, the adverse effects of waste charging schemes point to a need to formulate an effective policy that will prevent the outbreak of illegal waste disposal. Needless to say, illegal waste disposal is a sensitive attribute because it is a law-violating behavior. Therefore, if individuals are asked directly about their intention to dispose of waste illegally, they may under-report their true intention. To obtain a more comprehensive explanation of the intention of illegal waste disposal by identifying the factors driving such an intention, we employed structural equation modeling in which two sensitive questions related to intention were asked. The RRT in a multivariate analysis setting proved to be applicable in our practical environmental study. Municipal solid waste may include waste from residential and non-residential areas. To study individuals’ intentions of illegal waste disposal, we limited our scope to waste from residential areas. 

### 1.2. Theories Related to the Intention of Illegal Waste Disposal

To identify the factors involved in the illegal waste disposal intention, we adopted the theory of planned behavior (TPB) [20] and the general deterrence theory (GDT) [21] to construct our research model. 

The TPB explains the rational decision-making process of an individual. Under the TPB, an individual’s intention to commit a certain behavior is determined by that person’s subjective norms, attitude toward the behavior, and perceived behavioral control. Subjective norms are the perceived social pressures to conduct a behavior, which is the result of the perceived strength of the approval or disapproval from important reference people. Attitude toward a behavior refers to an individual’s favorable or unfavorable opinion toward the behavior, which results from his or her beliefs about the outcome or consequence of the behavior. Perceived behavioral control is a person’s perception of the ease or difficulty he or she would have in conducting a specific behavior or engaging in that behavior. The TPB has been applied to explain a wide range of pro-environmental behaviors, such as the use of public transportation [22,23], recycling behavior [24,25], food-waste reduction [26], and green purchase behavior [27]. In our study, we examined whether the three factors in the TPB affected individuals’ intentions of illegal waste disposal, and the resulting information provided us with some insights on policy promotion. 

The GDT suggests that punishment discourages individuals from conducting deviant behaviors [21,28]. Gibbs [21] provided empirical evidence demonstrating that the certainty and severity of legal sanctions reduce the incidence of crime. The certainty of sanctions is the probability that one will be punished after committing the deviant behavior, whereas severity of sanctions is the intensity of the punishment. Geerken and Gove [29] explicitly emphasized that the perceived certainty and severity of sanctions are more important than the actual certainty and severity. The GDT has been applied to explain the effect of punishment on various deviant or criminal behaviors, such as illegal disposal of construction and electrical waste [30], personal use of the Internet at work [31], and drinking and driving [32]. In determining the intention of illegal waste disposal, the GDT may provide implications for policy modification, such as to increase fines and reinforce monitoring.

### 1.3. Research Model and Hypotheses

**Figure 1:**
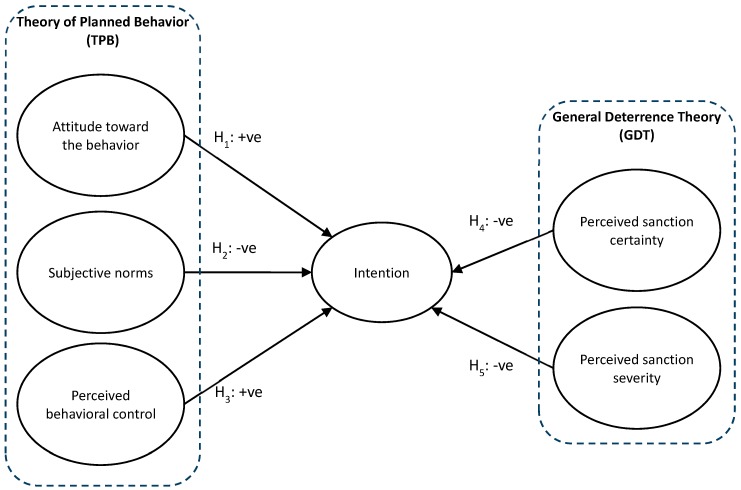
Research model and the hypotheses.

By incorporating the TPB and the GDT, we formed a research model for this study, as depicted in Figure 1. The corresponding hypotheses of this study were as follows:

H_1_: Attitude toward the behavior positively affects the intention of illegal waste disposal.
H_2_: Subjective norms negatively affect the intention of illegal waste disposal.
H_3_: Perceived behavioral control positively affects the intention of illegal waste disposal.
H_4_: Perceived certainty of sanctions negatively affects the intention of illegal waste disposal.
H_5_: Perceived severity of sanctions negatively affects the intention of illegal waste disposal.

### 1.4. Randomized Response Technique

The RRT is a classic surveying technique to encourage truthful responses toward sensitive topics through the addition of random noise to respondents’ answers during survey data collection [33]. This technique has been widely used in social science studies for nearly 50 years [7]. In this half century, the technique has also been modified or extended to different designs [3,34,35,36,37,38].

The RRT was originally proposed by Warner [2] to minimize the response bias from dichotomous questions about sensitive issues. Respondents would be randomly assigned to answer either the sensitive question or the inverse question with a “yes” or “no” answer [8]. As is shown in Table 1, the assignment undergoes a randomization procedure, such as rolling a die or whirling a spinner. The researcher would know only the probability for answering the sensitive questions but not the exact assignment results of each respondent. However, the use of inverse questions in Warner’s method limits the application of the RRT to a dichotomous sensitive question setting only, which may not be applicable to quantitative questions, such as the intention level and the frequency of particular behaviors.

Greenberg et al. [3] extended the RRT method by using an unrelated question design. As shown in Table 2, respondents would be asked to use a randomization device before answering the sensitive questions in the survey. Based on the assignment outcome, each of the respondents would be assigned to answer either a sensitive question on the concerned topic or a non-sensitive question on an unrelated topic. The assignment outcome would be known to the respondent only, thus the researchers would not know which question had been answered by the respondent. This procedure protects the respondents’ privacy and raises their confidence to answer sensitive questions truthfully. Different from Warner’s method, the use of UQD in this new RRT allows the RRT to be applied not only to dichotomous questions but also to quantitative questions. By defining different probabilities to the answers to the sensitive questions (e.g., drawing numbered cards or rolling a die in the example) and assessing the mean of the responses to the sensitive questions, the unknown population mean can be retrieved through mathematical execution.

Nevertheless, the classical RRT methods introduced by Warner [2] and Greenberg et al. [3] cannot estimate the covariances for examining the model involving latent variables. Therefore, an RRT in a multivariate analysis setting [37,38,39], which is an extension of the classic RRT method, can be applied to obtain high reliability estimates of the covariances among a set of sensitive and direct questions. However, the use of physical devices to administer randomization may hinder the respondents in answering the questionnaire.

This paper demonstrates the integration of the RRT and the SEM in a study using an online survey to examine the potential factors affecting people’s intention of illegal waste disposal. 

## 2. Materials and Methods 

### 2.1. Randomization Procedure through an Online Survey

With the combined popularity of the internet and the use of electronic devices, online surveys have become more effective and easier to operate, thus making their use a trend in health studies [40]. Traditionally, the RRT was conducted under a face-to-face interview so that physical devices such as cards, a coin, or dice could be used to generate randomized outcomes to determine whether the individual would answer the sensitive question or the unrelated question. Some scholars, such as Holbrook and Krosnick [41], have commented that using physical devices to generate randomization can be problematic because respondents may think the process is cumbersome and that might make them ignore the randomization procedure or drop out of the survey. Hence, using a physical randomization device, especially in an online survey, is not desirable. Online electronic devices such as a digital spinner [42] or an electronic coin toss [43] have been introduced in online surveys with RRT questions. In Peeters’s study [42], respondents were asked to answer either the sensitive question or the unrelated question solely based on the random outcomes of the digital spinner results generated by the computer (e.g., if the digital spinner stopped on an imprinted area, the person had to answer the sensitive question. If the spinner stopped on an empty area, the person was to answer the unrelated question). In Coutts and Jann [43], respondents were asked to answer either the sensitive question or the unrelated question solely on the basis of the random outcomes of the computer’s electronic coin tosses (e.g., if the electronic coin resulted in “head,” the respondent had to answer the sensitive question; if it was “tail,” the person was to answer the non-sensitive question). However, a shortcoming of using solely an online electronic device to execute the randomization procedure is that respondents may think the researcher is cheating by displaying a nonrandom outcome in which the researcher must surely know the true response from the respondent to the sensitive question. This discourages the respondents from giving honest answers. 

**Figure 2:**
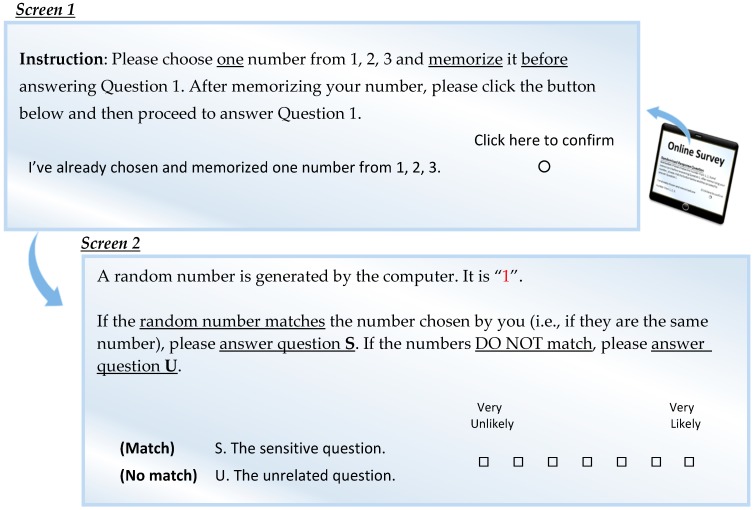
An example of this study’s randomization procedure.

To avoid that concern, our randomization procedure included an extra step that asked the respondents to select a private number in addition to the random outcome generated by the computer, and to answer the sensitive question only if the random number matched the respondent’s privately selected number. Under that procedure, the researcher had no opportunity to cheat by controlling the device and displaying a non-random number since he or she had no idea which number would be displayed nor which number the respondent had chosen. This procedure with the extra step increased the trustworthiness of our randomization device compared with that of Peeters [42] and that of Coutts and Jann [43]. An example of the randomization procedure we adopted is illustrated in Figure 2. 

### 2.2. Measures

The study’s self-administered online questionnaire was comprised of three parts: (1) the measurement items of the factors—attitude toward the behavior, subjective norms, perceived behavioral control, perceived severity of formal sanctions, and perceived certainty of sanctions, which are direct questions with no involvement of the RRT; (2) the measurement items of illegal waste disposal intention, which involved the RRT; and (3) general demographic information of the respondents. All of the measurement items in the research model had been validated in the literature and used a 7-point Likert scale. To ensure that our respondents could complete the questionnaire on their own without difficulties, pretesting [44] and a pilot study [45] were conducted before the actual data collection. The feedback from the pretest and pilot test showed that the questionnaire was clear and easy to understand, and we made only a few minor changes to the wording of the questions. The final set of measurement items is listed in Appendix A. 

Hong Kong’s waste charging scheme was still under proposal, thus citizens did not have solid ideas about the scheme. To provide a more holistic picture of the situation and to reduce response bias, we set up an explicit scenario that was based on the existing information about the waste-charging scheme. The scenario that we adopted in this study is also presented in Appendix A. 

### 2.3. Mathematical Execution behind the RRT

The concept of the RRT is to extract more trustworthy responses on sensitive issues by adding random noise into the data collection of the survey study. Under the UQD, a researcher obtains the observed responses pooled with the answers for sensitive and unrelated questions. The unknown means of the sensitive questions and the covariance structure among the sensitive and direct questions can be determined using the observed responses and the probability of the random assignments.

#### 2.3.1. Unrelated Question Design in a Likert Scale

To assess the mean value of intention of illegal waste disposal, we used the UQD in a Likert scale [37,38,39] in this study. An unrelated question was formed to pair with each sensitive question, as illustrated in Table 3. The randomization procedure described in Figure 2 was used for assigning each respondent to rate either question S (a sensitive question) or U (an unrelated question) without disclosing anything to the researcher. The respondent would answer on a 7-point Likert scale (from 1–“very unlikely” to 7–“very likely”) according to the question assigned from the randomization outcome. The researcher did not know whether the answer was for question S or question U.

#### 2.3.2. Estimation of the Response Mean for the Sensitive Question

Let *p* be the probability that a respondent will be assigned to answer question S. Let Z be the observed response pooled with the answers for randomly assigned questions S and U. The relationship between the response means $\mu_{Z}$, $\mu_{S}$, and $\mu_{U}$ can be derived as:

(1) $$\mu_{Z}=p\mu_{S}+\left(1-p\right)\mu_{U}.$$

As the UQD suggests, by dividing the whole sample into two subsamples (denoted by subsample 1 and subsample 2) and predefining the two different probabilities $p_{1}$ and $p_{2}$ of answering question S for each of the subsamples, the observed sample means ${\bar{Z}}_{1}$ and ${\bar{Z}}_{2}$ of Z in subsample 1 and subsample 2, respectively, form a set of simultaneous Equation (2):

(2) $$\{\begin{array}{c} {\bar{Z}}_{1}=p_{1}{\hat{\mu }}_{S}+\left(1-p_{1}\right){\hat{\mu }}_{U},\\ {\bar{Z}}_{2}=p_{2}{\hat{\mu }}_{S}+\left(1-p_{2}\right){\hat{\mu }}_{U}. \end{array}$$

By solving Equation (2) using the known ${\bar{Z}}_{1}$ and ${\bar{Z}}_{2}$ and the predefined $p_{1}$ and $p_{2}$, the desired estimated mean of the sensitive question ${\hat{\mu }}_{S}$ (and that of the unrelated question ${\hat{\mu }}_{U}$ as well) can be assessed.

#### 2.3.3. Estimation of the Response Variance for the Sensitive Question

The estimation of the response variance for the sensitive question shares the similar calculation procedure of the mean. The relationship among the response variances $\sigma_{Z}^{2}$, $\sigma_{S}^{2}$, and $\sigma_{U}^{2}$ can be expressed as:

(3) $$\left(\sigma_{Z}^{2}+\mu_{Z}^{2}\right)=p\left(\sigma_{S}^{2}+\mu_{S}^{2}\right)+\left(1-p\right)\left(\sigma_{U}^{2}+\mu_{U}^{2}\right).$$

Applying Equation (3) to subsample 1 and subsample 2 with predefined probabilities $p_{1}$ and $p_{2}$, the observed sample standard deviations ${\hat{\sigma }}_{z1}$ and ${\hat{\sigma }}_{z2}$ of Z in subsample 1 and subsample 2 forms a set of simultaneous equations:

(4) $$\{\begin{array}{c} \left({\hat{\sigma }}_{Z1}^{2}+{\hat{\mu }}_{Z1}^{2}\right)=p_{1}\left({\hat{\sigma }}_{S}^{2}+{\hat{\mu }}_{S}^{2}\right)+\left(1-p_{1}\right)\left({\hat{\sigma }}_{U}^{2}+{\hat{\mu }}_{U}^{2}\right),\\ \left({\hat{\sigma }}_{Z2}^{2}+{\hat{\mu }}_{Z2}^{2}\right)=p_{2}\left({\hat{\sigma }}_{S}^{2}+{\hat{\mu }}_{S}^{2}\right)+\left(1-p_{2}\right)\left({\hat{\sigma }}_{U}^{2}+{\hat{\mu }}_{U}^{2}\right). \end{array}$$

Note that ${\hat{\sigma }}_{Z}^{2}+{\hat{\mu }}_{Z}^{2}$ is equal to the sample mean of the squared response $\bar{ZZ}$, and Equation (4) can be modified to:

(5) $$\{\begin{array}{c} {\bar{ZZ}}_{1}^{}=p_{1}\left({\hat{\sigma }}_{S}^{2}+{\hat{\mu }}_{S}^{2}\right)+\left(1-p_{1}\right)\left({\hat{\sigma }}_{U}^{2}+{\hat{\mu }}_{U}^{2}\right),\\ {\bar{ZZ}}_{2}^{}=p_{2}\left({\hat{\sigma }}_{S}^{2}+{\hat{\mu }}_{S}^{2}\right)+\left(1-p_{2}\right)\left({\hat{\sigma }}_{U}^{2}+{\hat{\mu }}_{U}^{2}\right). \end{array}$$

The sample mean of the squared sample response ${\bar{ZZ}}_{1}^{}$ and ${\bar{ZZ}}_{2}^{}$ can be determined from the survey data, and the estimated means ${\hat{\mu }}_{s}$ and ${\hat{\mu }}_{U}$ can be assessed through the procedure in the previous part. Thus, the simultaneous Equation (5) can be solved to derive the estimated variances of the sensitive question ${\hat{\sigma }}_{S}^{2}$ (and of the ${\hat{\sigma }}_{U}^{2}$) that we want.

#### 2.3.4. Estimation of the Response Covariance for Two Sensitive Questions

To estimate the response covariance between two sensitive questions, the mean of their responses’ product is used. To demonstrate, let us use the second question on measuring illegal waste disposal, which is shown in Table 4, as an example.

Note that the probability that a respondent will be assigned to answer question S′ is also *p*. Let Z′ be the observed response combined by the answers for questions S′ and U′. Under the UQD method, the questions U and U′ have no relationship with the sensitive questions S and S′. Therefore, the relationship between the remaining response covariances $\sigma_{ZZ^{\prime }}^{}$, $\sigma_{SS^{\prime }}^{}$, and $\sigma_{UU^{\prime }}^{}$ can be expressed as:

(6) $$\sigma_{ZZ^{\prime }}^{}+\mu_{Z}\mu_{Z^{\prime }}=p\left(\sigma_{SS^{\prime }}^{}+\mu_{S}\mu_{S^{\prime }}\right)+\left(1-p\right)\left(\sigma_{UU^{\prime }}^{}+\mu_{U}\mu_{U^{\prime }}\right).$$

By using a similar approach to that in the previous section, we take the two subsamples, 1 and 2, with assigned probabilities $p_{1}$ and $p_{2}$, and form a set of simultaneous Equation (7) for the covariance $\sigma_{ZZ^{\prime }1}^{}$ and $\sigma_{ZZ^{\prime }2}^{}$ in the two subsamples:

(7) $$\{\begin{array}{c} \sigma_{ZZ^{\prime }1}^{}+\mu_{Z1}\mu_{Z^{\prime }1}=p_{1}\left(\sigma_{SS^{\prime }}^{}+\mu_{S}\mu_{S^{\prime }}\right)+\left(1-p_{1}\right)\left(\sigma_{UU^{\prime }}^{}+\mu_{U}\mu_{U^{\prime }}\right),\\ \sigma_{ZZ^{\prime }2}^{}+\mu_{Z2}\mu_{Z^{\prime }2}=p_{2}\left(\sigma_{SS^{\prime }}^{}+\mu_{S}\mu_{S^{\prime }}\right)+\left(1-p_{2}\right)\left(\sigma_{UU^{\prime }}^{}+\mu_{U}\mu_{U^{\prime }}\right). \end{array}$$

Note that $\sigma_{ZZ^{\prime }}^{}+\mu_{Z}\mu_{Z^{\prime }}$ is equal to the expected value or the mean of $ZZ^{\prime }$, and Equation (7) can be modified to:

(8) $$\{\begin{array}{c} {\bar{ZZ^{\prime }}}_{1}^{}=p_{1}\left({\hat{\sigma }}_{SS^{\prime }}^{}+{\hat{\mu }}_{S}{\hat{\mu }}_{S^{\prime }}\right)+\left(1-p_{1}\right)\left({\hat{\sigma }}_{UU^{\prime }}^{}+{\hat{\mu }}_{U}{\hat{\mu }}_{U^{\prime }}\right),\\ {\bar{ZZ^{\prime }}}_{2}^{}=p_{2}\left({\hat{\sigma }}_{SS^{\prime }}^{}+{\hat{\mu }}_{S}{\hat{\mu }}_{S^{\prime }}\right)+\left(1-p_{2}\right)\left({\hat{\sigma }}_{UU^{\prime }}^{}+{\hat{\mu }}_{U}{\hat{\mu }}_{U^{\prime }}\right). \end{array}$$

With the two sample means of the response product, ${\bar{ZZ^{\prime }}}_{1}^{}$ and ${\bar{ZZ^{\prime }}}_{2}^{}$, which are obtained from the survey data, we can solve the simultaneous Equation (8) and thereby determine the estimated covariance of the two sensitive questions ${\hat{\sigma }}_{SS^{\prime }}^{}$. In a multivariate analysis setting, and especially in structural equation modeling, multiple sensitive questions are usually required for measuring a latent sensitive trait. For that reason, the RRT with the UQD can contribute to this type of analytic design.

#### 2.3.5. Estimation of the Response Covariance for the Sensitive and Direct Questions

Let D be the observed response to a direct question in the survey questionnaire. Since the questions U and U′ also have no relationship with the direct question D, the relationship between the response covariance, $\sigma_{ZD}^{},$ and the covariance of S and D, $\sigma_{SD}^{},$ can be expressed as:

(9) $$\sigma_{ZD}^{}=p\sigma_{SD}^{}.$$

By dividing the whole sample into subsamples 1 and 2, as mentioned before, a pair of equations for the sample covariances ${\hat{\sigma }}_{ZD1}^{}$ and ${\hat{\sigma }}_{ZD2}^{}$ is formed.

(10) $$\{\begin{array}{c} {\hat{\sigma }}_{ZD1}^{}=p_{1}^{}{\hat{\sigma }}_{SD}^{},\\ {\hat{\sigma }}_{ZD2}^{}=p_{2}^{}{\hat{\sigma }}_{SD}^{}. \end{array}$$

Using the fact that $\sigma_{ZD}^{}+\mu_{Z}\mu_{D}$ is equal to the expected value of the mean of ZD, Equation (7) can be changed into:

(11) $$\{\begin{array}{c} {\bar{ZD}}_{1}^{}-{\bar{Z}}_{1}{\hat{\mu }}_{D1}=p_{1}^{}{\hat{\sigma }}_{SD}^{}\\ {\bar{ZD}}_{2}^{}-{\bar{Z}}_{2}{\hat{\mu }}_{D2}=p_{2}^{}{\hat{\sigma }}_{SD}^{} \end{array}\underset{}{\Rightarrow }{\hat{\sigma }}_{SD}^{}=\frac{{\bar{ZD}}_{1}^{}+{\bar{ZD}}_{2}^{}-{\bar{Z}}_{1}{\hat{\mu }}_{D1}-{\bar{Z}}_{2}{\hat{\mu }}_{D2}}{p_{1}^{}+p_{2}^{}}.$$

The estimates ${\hat{\mu }}_{D1}$ and ${\hat{\mu }}_{D2}$ are the sample means of D in subsample 1 and in subsample 2, respectively, and the sample means of the response product ${\bar{ZD}}_{1}^{}$ and ${\bar{ZD}}_{2}^{}$ can be calculated from each group of the subsample data. The estimated covariance of the sensitive question S and the direct question D, ${\hat{\sigma }}_{SD}^{}$, can then be determined using formula (11).

In our study, the randomization procedure let us define the probability $p_{1}^{}=$ 1/3 for subsample 1 and probability $p_{2}^{}=$ 2/3 for subsample 2. This randomization approach can be further adjusted by providing more options (e.g., “1, 2, 3, 4,” or “A, B, C, D, E”), but there must always be at least three choices. Otherwise, $p_{1}^{}=p_{2}^{}=$ 1/2 and the calculations above cannot be executed.

### 2.4. Data Collection and Analysis

The target respondents of the online survey were the general public over the age of 18 in Hong Kong. We identified the target respondents via a database collected from a market research firm, and we then sent them an e-mail invitation, an information sheet, and a hyperlink to the survey. Under the UQD, we had to divide the whole sample into two subsamples so that the mathematical execution mentioned before could be conducted to determine the statistics of illegal waste disposal intention. To ensure homogeneity in subsamples 1 and 2, we sent the same survey link to all of the target respondents, but the two versions of the survey were shown alternately (i.e., respondent 1 answered version 1 and respondent 2 answered version 2, and so on). Ultimately, we recruited 223 respondents (111 in sample 1 and 112 in sample 2) to participate in this study. When we conducted *t*-tests or chi-square tests on the demographic information of the two samples, we found no significant differences, thus indicating that the two samples were homogeneous. 

The RRT was performed by the software package R to estimate the covariance structure among the items for intention and the factors. The descriptive statistics of the items were also extracted through R. The covariance-based SEM was conducted using SPSS AMOS 22.0 for testing the five hypothesized paths in our research model.

## 3. Results

### 3.1. Descriptive Statistics

Our sample was diverse. Ninety two of the respondents were male (41.3%) and 131 were female (58.7%). The age of 37.2% of the respondents was 29 years old or younger, that of 48.0% was between 30 and 49 years old, and 14.8% of the respondents were 50 years old or older. Nearly three-quarters of the respondents were employed (74.9%), and the remaining respondents (25.1%) reported themselves to be homemakers, retired, or other.

The statistics of the measurement items were estimated as shown in Table 5. The intention of illegal waste disposal, which was derived by the RRT, was relatively low (the means were 2.98–3.04 from a possible maximum of 7). Unlike the values that would be obtained from direct sensitive questions, our mean value resulting from the RRT could provide more convincing and trustworthy estimates for illegal waste disposal intention among the general public in Hong Kong.

### 3.2. Structural Equation Model

**Figure 3:**
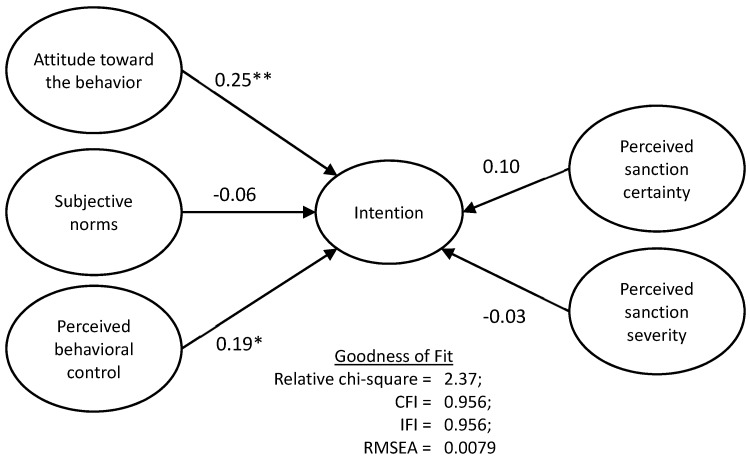
The results of the research model (* *p* < 0.05, ** *p* < 0.01). CFI = comparative fit index; IFI = incremental fit index; RMSEA = root mean squared error of approximation

The path coefficient and the analytic results are shown in Figure 3. The goodness-of-fit indices (relative chi-square = 2.37, CFI = .956, IFI = .956, RMSEA = .0079) showed that the overall model was acceptable. From the resulting model, two out of the five hypotheses were supported by statistically significant results. 

Attitude toward the behavior was found to positively affect the intention of illegal waste disposal (*β* = 0.25, *p* < 0.01), thus supporting H_1_. In addition, perceived behavioral control had a significant positive effect on the intention (*β* = 0.19, *p* < 0.05), thereby supporting H_3_. The findings thus indicated that subjective norms, perceived certainty of sanctions, and perceived severity of sanctions did not have any significant effects on the intention, meaning that H_2_, H_4_, and H_5_ were not supported.

The results of the SEM revealed that the intention of illegal disposal was significantly influenced only by the respondents’ attitudes toward illegal disposal and their perceived behavioral control. 

## 4. Discussion

In this study, we applied the RRT in an online survey to assess individuals’ intentions of illegal waste disposal. The topic is a sensitive one and cannot be easily examined through direct questioning. The research findings suggest that the average intention level of illegal waste disposal among the general public in Hong Kong remained at a low level, and the resulting structural equation model revealed that the respondents’ attitudes toward illegal disposal and perceived behavioral control were the factors influencing their intentions. Notably, subjective norms, perceived severity of formal sanctions, and perceived certainty of sanctions did not significantly affect the individuals’ intentions of illegal waste disposal. In other words, individuals tend to dispose waste illegally when their attitude toward such behavior is more favorable and/or when they perceive that they have more control of the behavior. Individuals may have had a favorable attitude toward illegal disposal due to a lack of awareness or ignorance about the environmental and/or legal consequences of the illegal municipal waste disposal [46,47]. To reduce this attitude, it is important for our government to raise the public awareness of proper waste disposal and their eco-responsibility through education. Similar to the strategy of anti-smoking, the government can emphasize the environmental and health hazards caused by illegal waste dumping through the printings of slogans on the designated rubbish bags. In term of perceived behavior control, when people were able to find suitable sites that made it easy to dispose of waste illegally, such as staircase, refuse rooms, and roadsides, their intentions increased. Our government should pay attention to this issue. They may consider redesigning the public rubbish bins and developing an efficient monitoring system to increase the difficulty of illegal waste disposal. Recently, surveillance camera systems have been installed in 80 blackspots in public places in Hong Kong for detecting illegal disposal of construction waste. Once the municipal waste charging is implemented, the government may consider expanding the blackspots’ coverage by investigating the potential public locations for illegal dumping of municipal waste. 

This study contributes to theory by applying the RRT to examine causal relationships among certain latent variables in which sensitive questions were involved and the topic was environmental and public health-related. Previous research had used RRT primarily in surveys with only one single dichotomous and sensitive question. Our study provides the steps and methodology for extending the RRT into a multivariate analysis setting, such as the SEM, in environmental and public health research. For ease of demonstration, we built a behavioral model with just two sensitive questions. However, similar steps and a comparable methodology could be applied to a behavioral model with more than two sensitive questions. The behavioral modeling and the findings in this study can serve as a useful starting point for developing a theoretical framework for research on illegal waste disposal in Hong Kong. As shown in Table 2, the continuous response of the behaviors can also be collected using the UQD design of RRT method. Therefore, future studies can investigate the actual behavior of illegal waste disposal among Hong Kong citizens after the waste charging scheme has been launched through the application of RRT. More importantly, the RRT approach can be extended to studies on other sensitive environmental and public health issues, such as drug abuse, sexual behavior, suicide risk, and smoking in private areas. Not limited to the continuous response, researchers can also use the RRT method introduced in this paper to investigate the relationship between the dichotomous response to the sensitive questions and other variables in the survey study.

The use of an online survey is also a key element in implementing the RRT. In addition to the technique’s advantages when compared with other traditional survey methods, such as face-to-face interviews or telephone interviews, it can facilitate the randomization procedure in RRT and ensure the privacy of the respondents. Besides automatically redirecting the respondents to work on the two different versions of the questionnaire, the subsampling procedure can be executed by splitting the sample into two subsamples randomly and then distributing the two versions of the questionnaire to the two subsamples via email (e.g., subsample 1–version 1; subsample 2–version 2) during the data collection [37]. However, researchers should be cautious about the homogeneity of the subsamples.

## 5. Conclusions

Research on environmental and public health topics sometimes involves sensitive questions, and if we ask those questions directly, we may receive dishonest responses. To reduce response distortion, the RRT is a useful and effective statistical method. Previous researchers have tended to apply the RRT in a single dichotomous question in a survey. Our RRT setting allows researchers to not only include multiple Likert-scale-rated or continuous sensitive questions in a survey but also to study their causal relationships. In the current study, we applied our innovative RRT to study individuals’ intentions of illegal waste disposal and found that their attitudes toward the behavior and their perceived behavioral control influenced such intentions. We expect that this study will help researchers to explore sensitive topics such as behavior in the environmental and public health fields.

## Figures and Tables

**Table 1:** Example of randomized response technique (RRT) application by using Warner’s method.

Probability	Assignment by Rolling a Dice ^1^	Question	Answer
23	1–4 → Sensitive Question:	I have an intention to dispose of waste illegally.	☐ Yes ☐ No
13	5–6 → Inverse Question:	I do not have any intention to dispose of waste illegally.	

^1^ Respondents would answer either a sensitive question or the inverse question, according to the outcome of rolling a die.

**Table 2:** Example of RRT application by using the unrelated question design (UQD).

Probability	Assignment by Rolling a Die^1^	Question	Answer
23	1–4 → Sensitive Question:	How many times did you illegally dispose of waste per week?	☐ ☐ ☐ ☐ ☐ 0 1–2 3–4 5–6 ≥7
13	5–6 → Unrelated Question:	How many times do you take a taxi per week?	

^1^ Respondents will answer either a sensitive question or the unrelated question, according to the outcome of rolling a die.

**Table 3:** Sample questions for assessing intention of illegal waste disposal using the UQD.

Question Type	Question	Answer						
S—Sensitive Question:	What is the likelihood that you would have disposed of waste illegally?	Very Unlikely		←─→			Very Likely	
U—Unrelated Question:	I will take Mass Transit Railway (MTR) tomorrow night.	1	2	3	4	5	6	7
		☐	☐	☐	☐	☐	☐	☐

**Table 4:** Sample questions for assessing intention of illegal waste disposal using the UQD.

Question Type	Question	Answer						
S′—Sensitive Question:	I could see myself disposing of waste illegally if I were in Taylor’s situation.	Strongly Disagree		←─→			Strongly Agree	
U′—Unrelated Question:	To me, taking vitamin pills everyday is healthy.	1	2	3	4	5	6	7
		☐	☐	☐	☐	☐	☐	☐

**Table 5:** The scale values and statistics of the measurement items in the survey questionnaire.

Latent Variable	Item	Scale (1–7)	Mean (SD)
Attitude toward the behavior	ATT1	(very bad—very good)	1.771 (1.173)
	ATT2	(very foolish—very wise)	2.457 (1.311)
	ATT3	(very bad unpleasant—very pleasant)	1.632 (1.103)
Subjective norms	SUB1	(strongly disagree—strongly agree)	5.744 (1.336)
	SUB2	(strongly disagree—strongly agree)	5.735 (1.341)
	SUB3	(strongly disagree—strongly agree)	5.722 (1.364)
Perceived behavioral control	PBC1	(strongly disagree—strongly agree)	3.830 (1.886)
	PBC2	(strongly disagree—strongly agree)	3.946 (1.902)
	PBC3	(strongly disagree—strongly agree)	3.942 (1.920)
Perceived severity of formal sanctions	PSS1	(strongly disagree—strongly agree)	4.462 (1.673)
	PSS2	(not severe at all—very severe)	3.906 (1.581)
Perceived certainty of sanctions	PCS1	(not severe at all—very severe)	4.081 (1.440)
	PCS2	(very low—very high)	3.803 (1.328)
Intention	INT1 ^a^	(very unlikely—very likely)	3.042 (1.976) ^b^
	INT2 ^a^	(strongly disagree—strongly agree)	2.980 (1.762) ^b^

a. The item was paired with an unrelated statement, under the UQD of the RRT; b. The mean and SD were estimated by the RRT.

## Appendix A: The Scenario and Items for ATT, SUB, PBC, PSS, PCS, and INT

**Scenario in the survey**

Taylor resides in a building in which the quantity-based waste-charging scheme has been implemented. For waste disposal, he needs to buy designated garbage bags from the property management, and to return the filled bags to a designated collection point. The money Taylor spends on the garbage bags is regarded as the waste-disposal charge. The property management strictly follows the scheme practice. On average, Taylor needs to spend a waste charge of HKD$50 per month for his family of 3. Taylor does not want to pay that charge. Thus, he tries to find unseen areas, street rubbish bins, or public refuse collection points in which to dispose of the waste illegally.

**Item**	**Item Description**	**Question Type**
Attitude toward the behavior [48]		
ATT1	Disposing of waste illegally is a very bad/very good idea.	Direct
ATT2	Disposing of waste illegally is a very foolish/very wise idea.	Direct
ATT3	Disposing of waste illegally is very unpleasant/very pleasant.	Direct
Subjective norms [49]		
SUB1	People who are important to me would think that I should not dispose of waste illegally. (strongly disagree—strongly agree)	Direct
SUB2	People whose opinions I value would think that I should not dispose of waste illegally. (strongly disagree—strongly agree)	Direct
SUB3	People who influence my behavior would think that I should not dispose of waste illegally. (strongly disagree—strongly agree)	Direct
Perceived behavioral control [50]		
PBC1	If I were Taylor, I would be able to find a site (e.g., an unseen area, street rubbish bin, or public refuse collection point) for easy illegal waste disposal. (strongly disagree—strongly agree)	Direct
PBC2	If I were Taylor, finding a site for illegal waste disposal would be entirely within my control. (strongly disagree—strongly agree)	Direct
PBC3	If I were Taylor, I would have the resources, the knowledge, and the ability to find a site for illegal waste disposal. (strongly disagree—strongly agree)	Direct
Perceived severity of formal sanctions [51]		
PSS1	If caught disposing of waste illegally by law enforcement officers, Taylor would be severely reprimanded. (strongly disagree—strongly agree)	Direct
PSS2	If caught disposing of waste illegally by law enforcement officers, Taylor’s punishment would be (not severe at all—very severe).	Direct
Perceived certainty of sanctions [51]		
PCS1	Taylor would probably be caught eventually by law enforcement officers, after disposing of waste illegally. (strongly disagree—strongly agree)	Direct
PCS2	The likelihood that law enforcement officers would discover that Taylor was disposing of waste illegally is (very low—very high)	Direct
Intention * [39]		
INT1	If you were Taylor, what is the likelihood that you would have disposed of waste illegally? (very unlikely—very likely) *(I will take Mass Transit Railway (MTR) tomorrow night.)*	RRT
INT2	I could see myself disposing of waste illegally if I were in Taylor’s situation. (strongly disagree—strongly agree) *(To me, taking vitamin pills everyday is healthy.)*	RRT
* *Statements in italics* are the unrelated and non-sensitive questions paired with their respective sensitive questions.		
